# The interaction of occupational stress, mental health, and cytokine levels on sleep in Xinjiang oil workers: A cross-sectional study

**DOI:** 10.3389/fpsyt.2022.924471

**Published:** 2022-09-28

**Authors:** Xue Li, Qiaoyun Xue, Xiaoting Yi, Jiwen Liu

**Affiliations:** ^1^Department of Public Health, Xinjiang Medical University, Urumqi, China; ^2^Department of Infection Management, The First Affiliated Hospital of Xinjiang Medical University, Urumqi, China

**Keywords:** occupational stress, mental health, sleep quality, cytokines, interaction

## Abstract

**Background:**

Sleep occupies one third of a person’s life, and good sleep quality is an important factor to ensure good health.

**Purpose:**

This study investigated and analyzed the occupational stress, mental health and sleep quality of oil workers, analyzed the effects of occupational stress and mental health on sleep, and explored the effects of the interaction between occupational stress, mental health and cytokines on sleep.

**Materials and methods:**

In this study, stratified cluster random sampling was used to conduct a cross-sectional survey on the occupational stress, mental health and sleep quality of 1,141 oil workers in the Occupational Health Examination Department of Karamay Central Hospital, from June 2019 to January 2020, and 30% of the participants were randomly selected for measurement of their cytokine levels: interleukin 2 (IL-2), interleukin 6 (IL-6), interleukin 8 (IL-8), and tumor necrosis factor α (TNF-α). The objectives were to analyze the effects of occupational stress and mental health on sleep quality, and to explore the effects of occupational stress, mental disorders and cytokine interactions on sleep.

**Results:**

There were 646 individuals (56.6%) who suffered from sleep disorders; the incidence of sleep disorders differed according to sex, age, professional title, working years, type of work and shift (*P* < 0.05). The scores for occupational stress, mental health, and sleep quality were positively correlated (*P* < 0.05). Multivariate logistic regression analysis showed that age (30–45 years) (OR = 1.753, 95% CI: 1.067–2.881), junior college and above (OR = 1.473, 95% CI: 1.025–2.118), borehole operation (OR = 2.689, 95% CI: 1.508–4.792), extraction of oil (OR = 2.405, 95% CI: 1.229–4.705), drilling (OR = 1.791, 95% CI: 1.017–3.155), shift work (OR = 2.144, 95% CI: 1.615–2.846), occupational stress (OR = 1.007, 95% CI: 1.002–1.013), and mental disorders (OR = 1.020, 95% CI: 1.009–1.032) are risk factors for sleep disorders. In the high occupational stress group and the mental disorders group as the control group, the results showed that the interaction between IL-2 and moderate occupational stress (OR = 0.778, 95% CI: 0.778–0.942), IL-2 and non-mental disorders (OR = 0.398, 95% CI: 0.398–0.468) were protective factors for the occurrence of sleep disorders. The results of structural equation modeling analysis showed that occupational stress and mental health had positive predictive effects on IL-2 level and sleep quality [normalized path coefficients (β) were 0.10\0.06\0.05\0.71, respectively]. Occupational stress had a direct positive predictive effect on mental disorder (β = 0.25), and sleep disorder had a direct negative predictive effect on IL-2 concentration (β = −0.21).

**Conclusion:**

Oil workers have serious sleep problems, so effective measures should be taken to reduce occupational stress and relieve mental health problems, and cytokine levels can be used as a predictor of sleep disorders.

## Introduction

Sleep is indispensable to human life activities: it is an essential factor in the maintenance of energy, and the maintenance and promotion of health. Good sleep not only protects cerebral cortical cells and replenishes their energy, but also regulates the balance of excitatory and inhibitory activities of the body, reduces fatigue, improves cognition and acceptance, improves mental health, and helps tissues and organs to work efficiently (1).

The term sleep disorders usually refers to acute or chronic prolonged sleep latency, excessive awakening in the sleep period, shortened sleep time, reduced sleep efficiency or poor subjective/objective evaluation of sleep quality (2). Sleep disorders are a serious problem, and chronic sleep deprivation can negatively affect a worker’s mood, health and ability to work. Long-term sleep disorders may lead to memory loss, slow reaction speed, lethargy and other effects, and may even lead to depression and suicidal tendencies, seriously affecting the quality of workers’ working lives (3, 4).

Occupational stress refers to harmful physical and psychological reactions that occur when job requirements do not match workers’ abilities, coping resources and demands (5). In recent years, both Chinese and foreign studies have shown that long-term stress will increase the number of nocturnal automatic responses of the body, thus reducing sleep quality (6, 7). When excessive work stress exceeds the body’s ability to regulate itself, it will lead to body function imbalance, resulting in the decline of sleep quality at night, causing insomnia, drowsiness and other sleep problems (8). Occupational stress will lead to job burnout, which will cause serious mental diseases such as anxiety and depression (9). The American Stress Association points out that 30–40% of illness absenteeism can be attributed to emotional and psychological disorders (10). At present, the ongoing coronavirus pandemic poses a serious threat to global mental health and seriously damages the physical and mental health of workers (11, 12). For professional people, many factors related to occupational stress have a significant negative impact on sleep, such as high work requirements, low work control (13), high effort and low return (14). Occupational stress is the main occupational risk factor that increases the risk of sleep problems (15).

Cell factors are an important part of the immune system, acting between the neuroendocrine system and the immune system, and allowing them to communicate with each other. The interaction medium is made up of immune cells and some non-immune cells that stimulate the synthesis and secretion of small proteins with a broad range of biological activities in the immune response, whose effects and adjustment play important roles in maintaining health and homeostasis. Interleukins (IL) are a class of cytokines produced by and used by a variety of cells, which include interleukin 2 (IL-2), interleukin 6 (IL-6), and interleukin 8 (IL-8). Their functions involve the expression and regulation of the immune response, acting on cells such as lymphocytes and macrophages. Studies have shown that sleep disorders such as sleep apnea, insomnia and REM sleep behavior disorder are associated with elevated levels of inflammatory molecules (16, 17). Given that inflammation can lead to fatigue, neuroinflammation from sleep disorders or insomnia may exacerbate fatigue in people with autoimmune diseases. Studies have found that IL-2 can significantly prolong the duration of slow-wave sleep in rats, suggesting that cytokines are involved in the regulation of sleep (18). Experimental studies in various mammals have shown that the pro-inflammatory cytokine tumor necrosis factor α (TNF-α) can enhance non-REM sleep when used in the peripheral or central nervous system (19). Vgontzas et al. (20) found that the diurnal concentration of IL-6 and TNF-α is related to chronic insomnia, thus explaining the daytime fatigue in patients with chronic insomnia.

Oil workers in Xinjiang generally work in the Remote Gobi Desert with a harsh natural environment. The long-term lack of family companionship and high-pressure working environment means that workers are prone to occupational stress and other mental health problems. In addition, the nature of shift work makes sleep problems more frequent. There are many reports on occupational stress and the mental health of oil workers, but there are few studies on the effects of occupational stress, mental health problems and cytokine interaction on sleep. Therefore, this study investigated and analyzed occupational stress, mental health, sleep quality and the level of cellular factors in Xinjiang oil workers, and explored the effect on sleep of their interactions.

## Materials and methods

### Participants

From June 2019 to January 2020, a stratified cluster random sampling method was adopted to select petroleum company workers who underwent physical examination in the Occupational Health Examination Department of Karamay Central Hospital of Xinjiang as the participants in this survey. After communicating with the management of the occupational disease physical examination department of the hospital before the survey, the list of employees of the company requiring physical examination was obtained. After the physical examination personnel were numbered, the study participants were randomly selected using the random number table method. Two large, medium and small oil companies (a total of six oil units) were selected, and 200 people were randomly selected from each company. Questionnaires were distributed to randomly selected participants on the day of their physical examination and returned after they were filled in. Inclusion criteria were:(1) employees aged 18–60 years with ≥1 year of service; (2) individuals who had not taken psychotropic drugs or sleeping drugs in the past year; (3) those who participated voluntarily in this survey. Exclusion criteria: (1) individuals who had retired; (2) those who have taken psychotropic drugs or sleep drugs in the past year; (3) those who refuse to participate in the survey. A total of 1,200 questionnaires were sent out in this survey, and 1,141 valid questionnaires were finally collected, with an effective questionnaire recovery rate of 95%.

In this study, a total of 646 people, accounting for 56.6% of the total, were screened for positive sleep disorders; 30% of the questionnaire respondents were randomly selected as the experimental research participants, giving 342 individuals. Therefore, 171 people were selected from the sleep disorder group and the control group, respectively, in this study, and 1:1 matching was adopted between those who were positive for sleep disorder and those who were negative for sleep disorder at age ±1 years old and of the same sex; the latter were selected as controls for a case–control study.

### Measurement of occupational stress

The Occupational Stress Inventory (OSI-R) developed by Osipow in was used for the questionnaire survey (21). It consists of three questionnaires: (1) the occupational role questionnaire (ORQ), including task overload, task discomfort, task ambiguity, task conflict, responsibility and work environment; (2) the personal strain questionnaire (PSQ), including business stress, psychological stress, interpersonal stress, and physical stress; (3) the personal resources questionnaire (PRQ), including leisure, self-care, social support, and rational handling indicators. Each questionnaire consists of 10 items, giving a total of 140 items, and each item is graded from 1 to 5. Higher ORQ and PSQ scores indicate heavier tasks and greater stress. Higher PRQ scores indicate a stronger ability to cope with stress. According to the scoring principle of the scale and with grouping by job characteristics, occupational stress intensity was evaluated from the ORQ questionnaire score, divided participants into a high stress group (total value > 160), a moderate stress group (120–160) and a low stress group (<120) (22). This scale has good internal consistency and reliability (Cronbach’s α = 0.59–0.86) (23).

### Measurement of mental health

This study used the Symptoms Checklist 90 (SCL-90) (24), which includes a total of 90 projects related to somatization, force, interpersonal relationships, depression, anxiety, hostility, terror, paranoia, psychosis and another 10 factors. The scale uses a 1–5 grade evaluation method: the higher the score, the more obvious the psychological symptoms. Mental disorder: total score is ≥160 or any factor score >2 (25). Symptom validity coefficients of the scale ranged from 0.77 to 0.99 (26).

### Measurement of sleep quality

This study used the Pittsburgh Sleep Quality Index (PSQI) compiled by Buysse et al. (27). The PSQI is used to evaluate sleep quality of in the last month, and is composed of 19 self-rated items and five other items. The 19th self-rated item and the five other items are not included in the score. The 18 remaining items are composed of seven parts, and each part is scored according to grades 0–3. The total score ranges from 0 to 21, with higher scores indicating poorer sleep quality. A PSQI score greater than seven indicates sleep disorder (28). This scale has good internal consistency and reliability (Cronbach’s α = 0.82–0.83) (29).

### Cytokine detection

The levels of IL-2, IL-6, IL-8, and TNF-α in the oil workers were determined by enzyme-linked immunosorbent assay (Shanghai Future Industry Co., Ltd., Shanghai, China). After collection, whole blood samples were centrifuged for 7 min (3000 RPM), and the supernatant stored at −80°C. Samples were transported at low temperature to avoid repeated freeze–thaw cycles.

### Quality control

(1) Epidemiological survey quality control: the investigators were trained before participating in the survey and were familiar with the questionnaire content and work processes. Before the survey, the researchers explained the content and answer method of the questionnaire in detail. The questionnaires were collected on the spot, and incomplete questionnaires were eliminated after collection.

(2) Laboratory quality control: blood samples were collected by nurses in the hospital. A pre-experiment and a follow-up experiment were carried out. The experimental steps were carried out in strict accordance with the instructions of the kit.

### Statistical analysis

The data were entered into the Epidata 3.0 database; SPSS version 22.0 software (SPSS Inc., Chicago, IL, USA) was used for the statistical analysis. If the measurement data followed a normal distribution and had uniform variance, $\bar{X}\pm S$ was used for description. A two independent samples *t*-test was used for comparison of means between two groups, and the χ^2^-test for comparison of rates, followed by Pearson correlation analysis. Multivariate analysis and interaction analysis for factors affecting sleep disorders were conducted by binary logistic regression analysis. Amos 25.0 software was used to establish the structural equation model and analyze the path coefficients between variables. The maximum likelihood method was used to estimate the model, and the final model was obtained after repeated iterative modification according to the modified index. Indirect effects were tested using the non-parametric percentile Bootstrap method with bias correction. When χ2/*df* ≤ 5.000, root mean square error of approximation (RMSEA) < 0.080, normalized Fit index (NFI), relative fit index (RFI), incremental fit index (IFI), Tucker-Lewis Index (TLI), and comparative fit index (CFI) were all >0.900, this indicated that all fitting indexes of the model were within the acceptable range. The significance level was α = 0.05.

## Results

### Demographic characteristics of oil workers

The demographic characteristics of the participants are shown in Table 1.

**TABLE 1:** Demographic characteristics of oil workers.

Variables	Group	Number (*n*)	Ratio (%)
Sex	Male	671	58.8
	Female	470	41.2
Age group, years	≤30	205	18
	30–45	619	54.3
	>45	317	27.8
Educational level	High school and below	859	75.3
	Junior College and above	282	24.7
Professional titles	No title	184	16.1
	Primary	153	13.4
	Secondary	277	24.3
	Senior	527	46.2
Working years	<5	180	15.8
	5–15	332	29.1
	>15	629	55.1
Type of work	Logging work	81	7.1
	Oil transportation	126	11
	Borehole operation	395	34.6
	Drilling	124	10.9
	Extract oil	415	36.4
Shift	Fixed day shift	354	31
	Shift	787	69
Marital status	Single	223	19.5
	Married	918	80.5
Monthly income	≤5,000	1000	87.6
	>5,000	141	12.4

### Occurrence of occupational stress, mental disorders and sleep disorders in oil workers with different demographic characteristics

The results showed that, among 1,141 oil workers, 109 (9.6%) had low occupational stress, 328 (28.7%) had moderate occupational stress, and 704 (61.7%) had high occupational stress. There were differences in occupational stress among workers of different ages, educational background, professional title, working years, shift work, and marital status (*P* < 0.05). In this survey, 553 people (48.5%) suffered from mental disorders, and the incidence of mental disorders was higher among oil workers with older age, higher education, and longer service life, and among oil transport workers, shift workers and married workers. In this survey, 646 individuals (56.6%) suffered from sleep disorders; the incidence of sleep disorders differed according to sex, age, professional title, working years, type of work, and shift situation (*P* < 0.05) (Table 2).

**TABLE 2:** Comparison of the incidence of occupational stress, psychological disorders and sleep disorders among oil workers with different demographic characteristics.

Variables	Group	Number	Occupational stress			Z/χ2	*P*	Mental disorders	χ2	*P*	Sleep disorders	χ2	*P*
			Low	Moderate	High								
Sex	Male	671	71 (10.6)	189 (28.2)	411 (61.3)	2.046	0.359	323 (48.1)	0.071	0.790	344 (51.3)	18.984	<0.001
	Female	470	38 (8.1)	139 (29.6)	293 (62.3)			230 (48.9)			302 (64.3)		
Age group, years	≤30	205	39 (19.0)	54 (26.3)	112 (54.6)	48.291	<0.001	76 (37.1)	105.109	<0.001	88 (42.9)	20.025	<0.001
	30–45	619	66 (10.7)	169 (27.3)	384 (62.0)			246 (39.7)			362 (58.5)		
	>45	317	4 (1.3)	105 (33.1)	208 (65.6)			231 (72.9)			196 (61.8)		
Educational level	High school and below	859	62 (7.2)	255 (29.7)	542 (63.1)	22.006	<0.001	449 (52.3)	20.134	<0.001	487 (56.7)	0.008	0.927
	Junior College and above	282	47 (16.7)	73 (25.9)	162 (57.4)			104 (36.9)			159 (56.4)		
Professional titles	No title	184	17 (9.2)	53 (28.8)	114 (62.0)	15.667	0.016	87 (47.3)	1.096	0.778	117 (63.6)	15.082	0.002
	Primary	153	27 (17.6)	41 (26.8)	85 (55.6)			73 (47.7)			71 (46.4)		
	Secondary	277	28 (10.1)	79 (28.5)	170 (61.4)			129 (46.6)			143 (51.6)		
	Senior	527	37 (7.0)	155 (29.4)	335 (63.6)			264 (50.1)			315 (59.8)		
Working years	<5	180	22 (12.2)	51 (28.3)	107 (59.4)	98.105	<0.001	70 (38.9)	37.128	<0.001	88 (48.9)	26.568	<0.001
	5–15	332	71 (21.4)	100 (30.1)	161 (48.5)			127 (38.3)			159 (47.9)		
	>15	629	16 (2.5)	177 (28.1)	436 (69.3)			356 (56.6)			399 (63.4)		
Type of work	Logging work	81	6 (7.4)	17 (21.0)	58 (71.6)	12.058	0.149	40 (49.4)	31.324	<0.001	30 (37.0)	33.089	<0.001
	Oil transportation	126	14 (11.1)	31 (24.6)	81 (64.3)			89 (70.6)			60 (47.6)		
	Borehole operation	395	31 (7.8)	108 (27.3)	256 (64.8)			172 (43.5)			261 (66.1)		
	Drilling	124	13 (10.5)	44 (35.5)	67 (54.0)			51 (41.1)			73 (58.9)		
	Extract oil	415	45 (10.8)	128 (30.8)	242 (58.3)			201 (48.4)			222 (53.5)		
Shift	Fixed day shift	354	59 (16.7)	115 (32.5)	180 (50.8)	39.480	<0.001	140 (39.5)	16.343	<0.001	160 (45.2)	27.247	<0.001
	Shift	787	50 (6.4)	213 (27.1)	524 (66.6)			413 (52.5)			486 (61.8)		
Marital status	Single	223	33 (14.8)	65 (29.1)	125 (56.1)	9.430	0.009	107 (48.0)	0.260	0.872	119 (53.4)	1.195	0.274
	Married	918	76 (8.3)	263 (28.6)	579 (63.1)			446 (48.6)			527 (57.4)		
Monthly income	≤5,000	1000	94 (9.4)	284 (28.4)	622 (62.2)	0.864	0.649	508 (50.8)	17.646	<0.001	567 (56.7)	0.023	0.880
	>5,000	141	15 (10.6)	44 (31.2)	82 (58.2)			45 (31.9)			79 (56.0)		

### Correlation analysis between occupational stress, mental health and sleep quality of oil workers

Pearson correlation analysis showed that the ORQ and PSQ were positively correlated with sleep quality (*P* < 0.05), and the PRQ was negatively correlated with sleep quality (*P* < 0.05). There was a positive correlation between mental health score and sleep quality (*P* < 0.05) (Table 3).

**TABLE 3:** Correlation analysis of occupational stress, mental health and sleep quality in petroleum workers.

Variables	Subjective sleep quality	Time of fall asleep	Sleeping time	Sleep efficiency	Sleep disorders	Hypnotic drugs	Daytime dysfunction	PSQI total score
ORQ score	0.164**	0.117**	0.117**	0.047	0.172**	0.108**	0.144**	0.219**
PSQ score	0.143**	0.180**	0.116**	0.066*	−0.053	0.094**	0.297**	0.245**
PRQ score	−0.066*	−0.090**	−0.074*	−0.037	0.009	−0.047	−0.099**	−1.115**
Somatization	0.104**	0.119**	−0.065*	0.032	0.114**	0.058*	0.073*	0.106**
Compulsive symptoms	0.070*	0.081**	−0.007	0.013	0.060*	0.074*	0.098**	0.100**
Interpersonal sensitivity	0.121**	0.084**	0.035	−0.006	0.031	0.134**	0.152**	0.147**
Depression	0.099**	0.063*	0.023	0.027	0.012	0.108**	0.173**	0.135**
Anxiety	0.173**	0.109**	0.014	0.015	0.179**	0.074*	0.058	0.149**
Hostility	0.030	0.031	−0.059*	−0.022**	−0.076**	0.069*	0.073*	0.019
Fear	0.086**	0.012	−0.008	−0.011	−0.046	0.026	0.088**	0.046
Paranoia	0.072*	0.011	0.001	0.035	−0.046	0.044	0.144**	0.075*
Psychosis	0.149**	0.126**	0.053	0.016	0.019	0.129**	0.176**	0.182**
Another factors	0.081**	0.057	0.013	−0.017	−0.016	0.079**	0.119**	0.089**
SCL-90 total score	0.140**	0.104**	0.004	0.015	0.048	0.114**	0.158**	0.154**

**P < 0.01, *P < 0.05.

### Logistic regression analysis of factors influencing sleep disorder in oil workers

With sleep quality as the dependent variable (0 = non-sleep disorder, 1 = sleep disorder), general demographic characteristics, occupational stress and mental disorders were included in the equation as independent variables, and binary logistic regression analysis was conducted. The analysis showed that age (30–45 years) (OR = 1.753, 95% CI: 1.067–2.881), college degree or above (OR = 1.473, 95% CI: 1.025–2.118), borehole operation (OR = 2.689, 95% CI: 1.508–4.792), drilling (OR = 2.405, 95% CI: 1.229–4.705), extraction of oil (OR = 1.791, 95% CI: 1.017–3.155), shift work (OR = 2.144, 95% CI: 1.615–2.846), occupational stress (OR = 1.007, 95% CI: 1.002–1.013), and mental disorders (OR = 1.020, 95% CI: 1.009–1.032) were risk factors for sleep disorders (Table 4).

**TABLE 4:** Logistic regression analysis of factors influencing sleep disorders in oil workers.

	*B*	SE	Wal*d*χ2	*P*	OR	95% CI.
Sex (Female)	0.232	0.146	2.515	0.113	1.261	0.947–1.678
Age (30–45)	0.561	0.253	4.909	0.027	1.753	1.067–2.881
Age (>45)	0.517	0.298	3.008	0.083	1.678	0.935–3.011
Educational level (Junior College and above)	0.388	0.185	4.379	0.036	1.473	1.025–2.118
Type of work (Oil transportation)	0.238	0.314	0.572	0.449	1.268	0.685–2.348
Type of work (Borehole operation)	0.989	0.295	11.245	0.001	2.689	1.508–4.792
Type of work (Drilling)	0.877	0.342	6.567	0.010	2.405	1.229–4.705
Type of work (Extract oil)	0.583	0.289	4.070	0.044	1.791	1.017–3.155
Working years (5–15)	−0.302	0.262	1.330	0.249	0.739	0.443–1.235
Working years (>15)	0.251	0.309	0.657	0.418	1.285	0.701–2.354
Shift	0.763	0.145	27.829	0.000	2.144	1.615–2.846
Marital status (Married)	−0.011	0.172	0.004	0.950	0.989	0.706–1.386
Monthly income (>5,000)	−0.029	0.195	0.021	0.884	0.972	0.663–1.424
Occupational stress	0.007	0.003	6.781	0.009	1.007	1.002–1.013
Mental disorders	0.020	0.006	11.928	0.001	1.020	1.009–1.032

### Comparison of cytokine concentrations in oil workers with different sleep quality scores

The concentration of IL-2 in oil workers in the sleep disorder group was lower than that in the group without sleep disorder, while the concentrations of IL-6 and TNF-α in oil workers with sleep disorder was higher than that in the group without sleep disorders, and the difference was statistically significant (*P* < 0.05) (Table 5).

**TABLE 5:** Comparison of cytokine concentrations in oil workers with different sleep quality scores.

Variables	Non-sleep disorders	Sleep disorders	*t*	*P*
IL-2	4.48 ± 1.12	4.11 ± 1.14	3.045	0.003
IL-6	30.52 ± 6.54	32.58 ± 7.21	−2.056	0.040
IL-8	102.31 ± 15.35	101.35 ± 18.50	0.522	0.602
TNF-a	74.12 ± 12.10	76.64 ± 10.50	−2.056	0.040

### Effects of occupational stress, mental disorders and cytokines on sleep in oil workers

With sleep quality as the dependent variable (0 = non-sleep disorder, 1 = sleep disorder), occupational stress, mental disorder and cytokines significant in the univariate analysis (Table 4) were included in the equation for binary logistic regression analysis. The results showed that occupational stress (OR = 1.037, 95% CI: 1.020–1.054) and mental disorder (OR = 1.047, 95% CI: 1.037–1.057) were risk factors for the occurrence of sleep disorder. The more serious the occupational stress and mental disorder, the higher the risk of sleep disorder. IL-2 (OR = 0.616, 95% CI: 0.433–0.876) was a protective factor for sleep disorders, and the higher the concentration of IL-2, the lower the risk of sleep disorders (Table 6).

**TABLE 6:** Logistic regression analysis of the effects of occupational stress, mental disorders and cytokines on sleep disorders in oil workers.

Variables	*B*	SE	Waldχ^2^	*P*	OR	95% CI.
Occupational stress	0.036	0.008	18.735	<0.001	1.037	1.020–1.054
Mental disorders	0.046	0.005	84.399	<0.001	1.047	1.037–1.057
IL2	−0.485	0.18	7.27	0.007	0.616	0.433–0.876
IL6	0.021	0.03	0.521	0.470	1.022	0.964–1.083
TNF-a	0.001	0.015	0.007	0.935	1.001	0.973–1.031

### Effects of occupational stress, mental health and cytokine interactions on sleep in oil workers

Based on the above multivariate logistic regression analysis of sleep disorders, the effects of occupational stress, mental disorder and cytokine interactions on sleep disorders were analyzed. The group with high occupational stress and mental disorder were taken as the reference group. The effects of IL-2 × low occupational stress, IL-2 × moderate occupational stress and IL-2 × non-mental disorders on sleep disorders were analyzed. The results showed that IL-2 × moderate occupational stress (OR = 0.778, 95% CI: 0.778–0.942) and IL-2 × non-mental disorders (OR = 0.398, 95% CI: 0.398–0.468) were protective factors for the occurrence of sleep disorders when compared with high occupational stress and mental disorders (Table 7).

**TABLE 7:** Effects of occupational stress, psychological health and cytokine interactions on sleep in oil workers.

	*B*	SE	Waldχ^2^	*P*	OR	95% CI.
IL2*low occupational stress	−0.162	0.405	0.161	0.688	0.850	0.850–1.879
IL2*moderate occupational stress	−0.251	0.098	6.624	0.010	0.778	0.778–0.942
IL2*non-mental disorders	−0.922	0.083	122.225	<0.001	0.398	0.398–0.468

*Means interaction.

### Pathway analysis of occupational stress, mental health, cytokines and sleep

**FIGURE 1:**
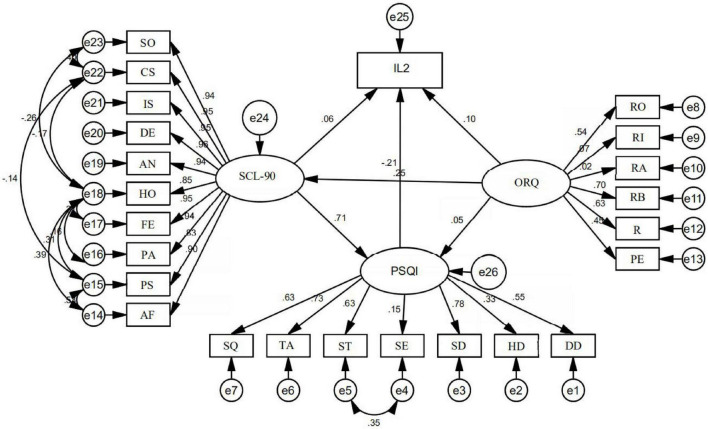
Occupational stress–mental health–cytokine–sleep pathway analysis. IL2, interleukin-2; SCL-90, mental health; ORQ, occupational stress; PSQI, sleep quality; SO, somatization; CS, compulsive symptoms; IS, interpersonal sensitivity; DE, depression; AN, anxiety; HO, hostility; FE, fear; PA, paranoia; PS, psychosis; AF, another factor; SQ, subjective sleep quality; TA, time of falling asleep; ST, sleeping time; SE, sleep efficiency; SD, sleep disorders; HD, hypnotic drugs; DD, daytime dysfunction; RO, role overload; RI, role insufficiency; RA, role ambiguity; RB, role boundary; R, responsibility; PE, physical environment.

The results of the structural equation analysis showed that the χ^2^/*df* = 2.638, RMSEA = 0.069, NFI = 0.916, RFI = 0.902, IFI = 0.946, TLI = 0.937, CFI = 0.946: all fitting indexes were within the acceptable range, indicating that the model fitted well. Occupational stress and mental health had positive predictive effects on IL2 level and sleep quality [the normalized path coefficient (β) was 0.10 and 0.06, 0.05, and 0.71, respectively], indicating that the higher the occupational stress level and the more serious the psychological disorder, the higher the IL2 concentration, and the worse the sleep quality. In addition, occupational stress had a direct positive predictive effect on psychological disorders (β = 0.25), indicating that the higher the level of occupational stress, the more serious the psychological disorder. Sleep disturbance had a direct negative predictive effect on IL2 concentration (β = −0.21), indicating that the worse the sleep quality, the lower the IL2 concentration (Figure 1). The total effects, direct effects and indirect effects among variables are shown in Table 8.

**TABLE 8:** Total effects–direct effects–indirect effects among variables.

	Total effect	direct effect	indirect effect
Occupational stress–mental health	0.249	0.249	–
Occupational stress–IL2	0.072	0.104	−0.032
Occupational stress–Sleep disorders	0.225	0.049	0.176
mental health-IL2	−0.088	0.057	−0.145
mental health-sleep disorders	0.706	0.706	–
Sleep disorders-IL2	−0.205	−0.205	–

## Discussion

Sleep is closely related to people’s health and is an important prerequisite to ensure the normal working of the body. For professional people in particular, a good sleep can make them energetic, with higher work efficiency. Therefore, identifying the risk factors that may affect sleep can provide some help in improving the sleep quality of workers. A previous study on the sleep of oil workers found that age and shift work were the factors influencing sleep disorders, but this study focused on analyzing the mediating effect of sleep disorders in the influence of gender and age on hypertension, and did not conduct an in-depth study on the factors influencing sleep disorders in oil workers (30). Therefore, this study focused on the factors affecting sleep in oil workers, and further analyzed the relationship between cytokine levels and sleep.

The study found that the incidence of sleep disorders varied by age, education, shift work and type of work. This is the same conclusion as some previous studies (31–33). In addition to population characteristics as factors affecting sleep, occupational stress and mental health also increase the risk of sleep disorders. Occupational stress is common among professional people; moderate stress can improve the efficiency of workers, but long-term high stress will compromise mental health, resulting in anxiety, depression and other negative emotions, and the accumulation of negative emotions will precipitate mental health problems (34). The study demonstrated that the more serious the occupational stress and psychological disorder, the greater the impact on sleep (31). Poor sleep quality at night will lead to insufficient energy for workers to complete their work during the day and further adversely affect their mental health status (31). Both here and abroad, study has found that occupational stress is a risk factor for sleep quality, and with the increase of occupational stress, sleep quality will be poorer (35, 36). Kploanyi et al. (37) investigated 235 telecom employees and found that employees with excessive occupational pressure reported a higher risk of insomnia. Knudsen et al. (38) also believe that work pressure is closely related to sleep quality, and that high work pressure leads to poor sleep quality, difficulty in falling asleep, and difficulty in maintaining sleep. In a study of nurses, Mark et al. (39) found that higher job requirements and stress levels were associated with higher levels of anxiety and depression, while social support, reward and mental health problems were negatively correlated. Tatsuse et al. (40) also found that sleep disorders are related to mental health and stress. Previous studies only verified the association between occupational stress, mental health and sleep, but did not clarify the effect size of the three. In this study, we identified the total effects, direct effects and indirect effects among variables through path analysis.

The study has found that there is a relationship between immune function and sleep, and cellular immune factors may be involved in sleep regulation (41). IL-2 is mainly produced by activated T lymphocytes, and its main biological activities are to promote the proliferation of T lymphocytes and NK cells, participate in the immune response and immune regulation of the body, and produce different biological effects by binding different receptor subtypes (42). Steel et al. (43) found that IL-2 plays a mediating role in the relationship between sleep and survival rate by studying the relationship between cytokine regulation of sleep and survival rate in cancer patients. Wang et al. (44) reported that the IL-2 level in the brains of workers with sleep disorder was lower than that of the non-sleep disorder group, which is consistent with the results of this study. In a previous study on the relationship between occupational stress and immune factors, it was found in the relationship between occupational stress and immune factors that occupational stress was related to IL-2 level, and the authors reported that the IL-2 level of the occupational stress group was significantly lower than that of the control group (45). This is different from the conclusion of this study, which found that occupational stress had a direct positive predictive effect on IL2, the total effect was 0.072, the direct effect was 0.104, but the indirect effect with sleep disorder as the mediating variable was −0.032, which needs to be confirmed by further research. As a chronic stressor, occupational stress will affect the body’s ability to regulate itself and lead to body function imbalance, resulting in a series of sleep problems such as insomnia and decreased sleep quality (13, 46). This study has demonstrated that long-term occupational stress or other negative emotions in workers will lead to the occurrence of psychological disorders, and subsequently effect changes in neuroendocrine levels in the body, resulting in changes in cytokine levels, and increase the risk of sleep problems (47).

## Conclusion

This research identifies that, in addition to demographic characteristics, occupational stress, mental health and cytokine levels also affect the sleep quality of oil workers. Occupational stress and mental disorders were risk factors for sleep disorder, and that the level of IL-2 was a protective factor for sleep disorder. The lower the IL-2 level, the higher the risk of sleep disorder. Low occupational stress and the interaction between mental health and IL-2 may reduce the risk of sleep disorders.

### Implications

This study provides a reliable basis to help employers to develop strategies to improve workers’ mental health and sleep quality. It is suggested that employers should reduce the stress suffered by workers and improve their mental health to reduce the occurrence of sleep problems. In this study, cytokine levels were used as sleep-related predictors to evaluate workers’ sleep quality.

### Limitations

The disadvantages of this study are as follows: First, as a special occupational group, oil workers have a higher labor intensity and a more difficult working environment than general workers. Therefore, the conclusions of this study need to be further verified in other occupational groups. Second, this study is a cross-sectional study, so it is difficult to infer causality. Finally, a total of four cytokines were measured in this study, but only IL-2 was associated with sleep according to the multivariate analysis. However, the other three cytokines were also associated with sleep in other studies. Therefore, the relationship between these cytokines and sleep will be further investigated in subsequent studies.

## Data availability statement

The original contributions presented in this study are included in the article/supplementary material, further inquiries can be directed to the corresponding author.

## Ethics statement

This study was approved by the Ethics Committee of Xinjiang Medical University. The patients/participants provided their written informed consent to participate in this study.

## Author contributions

XL, QX, and XY contributed to the acquisition, analysis, and interpretation of data. All authors contributed substantially to the work presented in this manuscript, were involved in drafting the manuscript and revising it for important intellectual content, and reviewed and approved the final manuscript. XL, QX, and JL conceived and designed the study.
